# Diffuse Gliomas with *FGFR3*::*TACC3* Fusion: Morphological and Molecular Features and Classification Challenges

**DOI:** 10.3390/cancers16091644

**Published:** 2024-04-25

**Authors:** Elena Marastoni, Davide Mulone, Valeria Barresi

**Affiliations:** Department of Diagnostics and Public Health, University of Verona, 37134 Verona, Italy; elena.marastoni@studenti.univr.it (E.M.); davide.mulone@studenti.univr.it (D.M.)

**Keywords:** *FGFR3*::*TACC3*, FGFR3, glioma, PLNTY, glioblastoma, DNA methylation, ganglioglioma

## Abstract

**Simple Summary:**

*FGFR3*::*TACC3* fusion is a driver, potentially targetable, alteration detected in approximately 4% of diffuse gliomas. Diffuse gliomas with *FGFR3*::*TACC3* fusion (F3T3 gliomas) and high-grade histological features harbor molecular stigmata and the DNA methylation profile of glioblastoma, though they are associated with slightly longer patient survival. Histologically low-grade F3T3 gliomas are molecularly heterogeneous and likely comprise three epigenetic groups. One includes tumors, exclusive to adults, displaying genetic and epigenetic features of glioblastoma and potentially representing precursors of high-grade gliomas. The second group lacks the molecular features of glioblastoma and has an epigenetic profile similar to that of dysembryoplastic neuroepithelial tumors. Finally, tumors in the third group are epigenetically close to gangliogliomas. Owing to their genetic and epigenetic heterogeneity, F3T3 gliomas do not represent a distinct nosological entity. Further research is needed to clarify the prognosis, refine the grading, and determine the optimal treatment approaches for these tumors.

**Abstract:**

*FGFR3*::*TACC3* fusion is a driver, potentially targetable, genetic alteration identified in approximately 4% of high-grade diffuse gliomas and rare cases with low-grade histology. Herein, we review the genetic and epigenetic features of these tumors and highlight the challenges in their classification and grading. Diffuse gliomas with *FGFR3*::*TACC3* fusion display unique histopathological and molecular features, including an oligodendroglioma-like appearance, calcifications, and CD34 extravascular immunoreactivity. High-grade tumors exhibit molecular alterations and a DNA methylation profile typical of glioblastoma, suggesting that they may represent a subtype clinically characterized by a slightly better prognosis. Tumors with low-grade morphology are genetically and epigenetically heterogeneous. Some, exclusive to adults, have molecular alterations typical of glioblastoma, although most do not match any methylation classes, using version 12.5 of the Heidelberg classifier. Another group, which mostly affects children or adolescents, lacks the molecular features of glioblastoma and has a DNA methylation profile similar to that of low-grade glioneuronal tumors. In conclusion, diffuse gliomas with *FGFR3*::*TACC3* fusion do not constitute a distinct nosological entity, owing to their genetic and epigenetic diversity. Further studies are warranted to clarify the biological aggressiveness of tumors with low-grade histology to refine the grading and determine the optimal treatment strategy.

## 1. Introduction

Gliomas account for approximately 26% of all central nervous system (CNS) tumors [1]. They are currently classified according to the fifth edition of the World Health Organization (WHO) classification of CNS tumors, which was published in 2021 (WHO 2021) [2]. Prior to the WHO 2021 classification, the diagnosis of gliomas was largely reliant on tumor histology, leading to considerable interobserver variability. However, tumors with similar morphology can exhibit different molecular features, which are more closely associated with biological and clinical aggressiveness than histopathology. Therefore, the WHO 2021 classification incorporates both molecular and histological characteristics as diagnostic criteria for gliomas, enabling a more comprehensive diagnostic approach.

According to the WHO 2021 classification, gliomas are distinguished into diffuse, which have an infiltrative growth pattern, and circumscribed, which harbor an expansive growth pattern [2]. They are graded into four grades of malignancy, with grade 1 being the most indolent and grade 4 being the most aggressive [2]. Diffuse gliomas are further categorized into “adult-type”, “pediatric-type” low-grade, and “pediatric-type” high-grade. The former mainly affect adults, whereas “pediatric-type” diffuse gliomas are more common in children and young adults. However, both types can affect patients of any age group. Based on the mutational status of *IDH1/2* and the presence of chromosome 1p and 19q deletion, adult-type diffuse gliomas are classified as astrocytoma *IDH*-mutant (grades 2, 3, or 4), oligodendroglioma *IDH*-mutant and 1p/19q codeleted (grades 2 or 3), and glioblastoma (GBM) *IDH*-wildtype (grade 4) [2]. The latter has the worst prognosis, with a median survival of only 8 months [1]. It is defined as a diffuse astrocytic tumor, which lacks mutations in *IDH1/2* and in *H3*, and shows one or more of the following histological or genetic features: microvascular proliferation, necrosis, *TERT* promoter *(pTERT)* mutation, *EGFR* gene amplification, and +7/−10 chromosome copy number changes [3]. Nevertheless, the GBM *IDH*-wild type represents a heterogeneous group of tumors, sharing the absence of mutations in *IDH1/2* and *H3*, which can be further dissected.

Pediatric-type low-grade diffuse gliomas comprise four different types: diffuse astrocytoma *MYB* or *MYBL1*-altered (grade 1), angiocentric glioma (grade 1), diffuse low-grade glioma with Mitogen-Activated Protein Kinase (MAPK) pathway alterations (no definitive grade assigned by WHO 2021), and polymorphous low-grade neuroepithelial tumor of the young (PLNTY) (grade 1) [2,4]. Finally, pediatric-type high-grade diffuse gliomas include diffuse midline glioma *H3* K27-altered (grade 4), diffuse hemispheric glioma *H3* G34-mutant (grade 4), diffuse pediatric-type high-grade glioma *H3* and *IDH*-wildtype (grade 4), and infant-type hemispheric glioma (no definite grade assigned by the WHO 2021) [2].

The Fibroblast Growth Factor Receptor (FGFR) family comprises five distinct members, including four membrane-bound tyrosine kinase receptors (RTKs), FGFR1, FGFR2, FGFR3, and FGFR4, and a kinase-lacking coreceptor, FGFR5 or FGFRL1 [5]. They can bind to various ligands (FGF, Cadherins, Nectins, Neuroplastin, NCAMs, L1-CAM, Neurexins, Ig-LON, FLRTs, Integrins) through their extracellular membrane domains [6]. *FGFR* gene alterations, including amplification, mutations, and rearrangements, have been detected in several types of tumors, with a reported frequency ranging between 7 and 9.2% [7,8,9]. Rearrangements represent approximately 10% of all *FGFR* alterations in cancer, with *transforming acidic coiled coil containing the protein 3 (TACC3)* being the most frequent fusion partner [9]. The latter encodes a centrosomal protein with coiled-coil domain, which is involved in mitosis [10]. Notably, *FGFR3*::*TACC3* fusion has been found in approximately 4% of diffuse gliomas with morphological features consistent with GBM *IDH*-wildtype and in 4% of diffuse gliomas with low-grade histology [11,12]. Diffuse gliomas with *FGFR3*::*TACC3* fusion (F3T3 gliomas) may potentially be treated with FGFR inhibitors [13]. Therefore, their identification may be relevant for therapeutic purposes.

In this review, we summarize the histopathological and molecular features of F3T3 gliomas, the current challenges in the diagnosis and classification of these tumors, and methods for detecting *FGFR3*::*TACC3* fusion in tumor tissue.

## 2. *FGFR3*::*TACC3* Fusion

*FGFR3* and *TACC3* are located 48 kb apart on human chromosome 4p16 [14]. The *FGFR3*::*TACC3* fusion gene results from tandem duplication and inversion of a 70 kb region on 4p16.3 [15], with at least fourteen possible rearrangements between *FGFR3* and *TACC3*, depending on different breakpoints in the two genes. The most frequent is the *FGFR3ex17-TACCex11* rearrangement, followed by *FGFR3ex17-TACC3ex10* and *FGFR3ex17-TACC3ex8* [16]. Despite the high variation among the breaking points of the FGFR3-TACC3 fusion protein, the intracellular tyrosine kinase domain of FGFR3 is fused upstream of the coil-coiled domain at the C-terminus of TACC3 in all cases [17]. The FGFR3-TACC3 fusion protein has a driver role in glioma oncogenesis. Indeed, astrocytes transfected with this protein can grow in anchorage-independent conditions and form gliomas if injected into immunodeficient mice [14]. Although the mechanisms by which the FGFR3-TACC3 protein induces gliomagenesis have not been fully clarified, in vitro studies have shown that this protein displays constitutive, ligand-independent phosphorylation of the tyrosine kinase domain in FGFR3 and induces aneuploidy [14]. Indeed, owing to an arc-shaped configuration, the FGFR3-TACC3 protein encases the metaphase spindle poles with an asymmetry towards one of the poles during mitosis, thus generating aneuploidy [14]. The oncogenic potential of this fusion seems also related to the activation of oxidative phosphorylation and mitochondrial metabolism [18].

As it is an oncogenic driver, is clonal, and is stable over recurrence, the FGFR3-TACC3 protein represents an attractive therapeutic target for gliomas. However, trials testing the efficacy of FGFR inhibitors in patients with F3T3 gliomas have demonstrated only moderate efficacy in comparison to the remarkable results obtained in other cancers harboring the *FGFR3*::*TACC3* fusion [13]. A potential reason for this could be the difficulty in these drugs in permeating the blood–brain barrier [13].

Given that *FGFR3* and *TACC3* are located in close proximity, on 4p 16.3 [14], detection of the fusion using Fluorescent In Situ Hybridization (FISH) assay is not feasible [19]. The gold standard for detecting this fusion is either targeted RNA-sequencing or whole transcriptome sequencing. Additionally, RT-PCR on either frozen or formalin-fixed and paraffin-embedded samples, as well as next-generation sequencing covering the intronic regions of the two genes involved in the fusion, can be used [16,20,21,22]. In a study, next-generation sequencing demonstrated higher sensitivity in identifying *FGFR3*::*TACC3* fusions in gliomas than RT-PCR performed on frozen tissue [23].

Assessment of FGFR3 immunohistochemical expression represents a useful screening tool for the identification of F3T3 gliomas with *FGFR3*::*TACC3* fusion [20,24]. Indeed, tumors harboring this genetic alteration overexpress FGFR3 owing to the loss of a specific sequence in the 3′-untranslated region of FGFR3, which is essential for gene regulation by miR-99 [21]. According to published studies, the immunohistochemical overexpression of FGFR3 predicts *FGFR3*::*TACC3* fusion with a sensitivity of 92–100% and a specificity of 86–100% [16,20,23].

## 3. Histopathological Features of Diffuse Gliomas with *FGFR3*::*TACC3* Fusion

**Figure 1:**
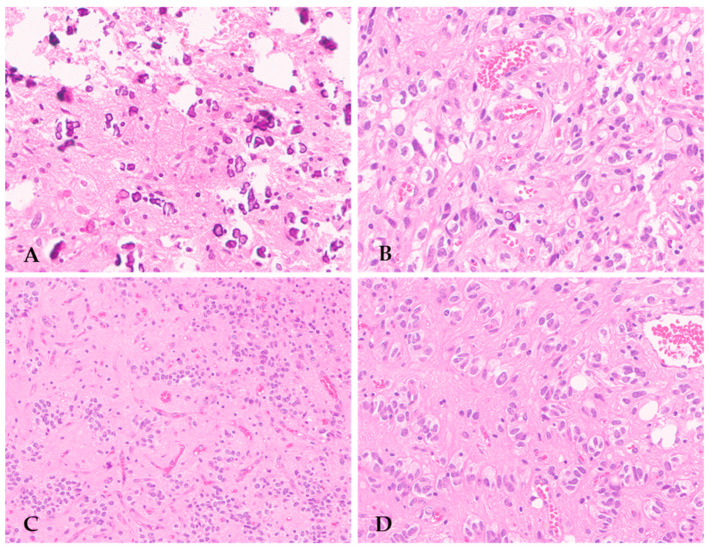
Recurring peculiar morphological features of F3T3 gliomas, including numerous calcifications (**A**) (original magnification, ×200), oligodendroglial-like cells with clear perinuclear halo (**B**) (original magnification, ×200), perivascular arrangement resembling pseudorosettes of ependymoma (**C**) (original magnification, ×100), and nuclear palisading (**D**) (original magnification, ×200).

The majority of F3T3 gliomas display high-grade histopathological features, including brisk mitotic activity, microvascular proliferation, and/or necrosis, consistent with GBM *IDH*-wildtype morphology [20]. More rarely, they lack these histological features of malignancy and appear as low-grade diffuse gliomas [20]. However, both low- and high-grade F3T3 gliomas show distinctive recurrent histopathological features, which are useful clues for their identification in routine practice. In a cohort of 30 cases, Bielle et al. first noticed that F3T3 gliomas displayed recurring morphological features, consisting of calcifications, an endocrinoid vascular pattern formed by thin and ramified vessels, oligodendroglial-like tumor cells with ovoid and uniform nuclei and a clear peri-nuclear halo, nuclear palisading, and desmoplasia (Figure 1) [20].

These authors also highlighted that a proportion of F3T3 gliomas may show a peri-vascular arrangement of tumor cells with interposed anuclear zones, simulating the pseudorosettes of ependymoma [20] (Figure 1C). Subsequent studies have confirmed the presence of these recurrent histopathological features in other cohorts of F3T3 gliomas, strengthening the association between morphology and genetics [12,25,26,27,28]. Notably, high-grade F3T3 gliomas had a significantly lower mitotic rate and a higher vascular density than GBM *IDH*-wildtype devoid of the *FGFR3*::*TACC3* fusion [20].

**Figure 2:**
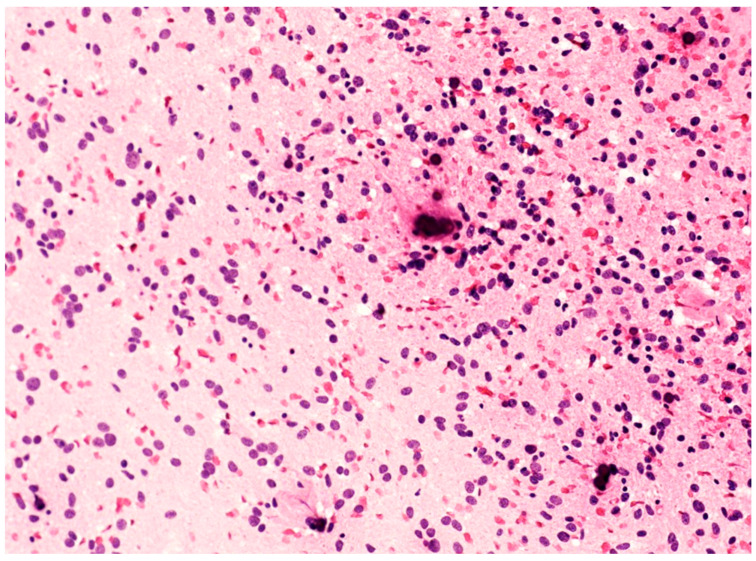
Cytological smear of an F3T3 glioma, showing monotonous oligodendroglial-like cells with roundish/ovoid nuclei and calcifications (original magnification, ×200).

The peculiar histopathological aspects of F3T3 gliomas can also be observed in cytological smears during intra-operative examination (unpublished data). In this setting, a diffuse glioma showing numerous calcifications and relatively monotonous cells with an oligodendroglial-like appearance and ovoid nuclei (Figure 2) should prompt consideration of the possibility of F3T3 glioma.

**Figure 3:**
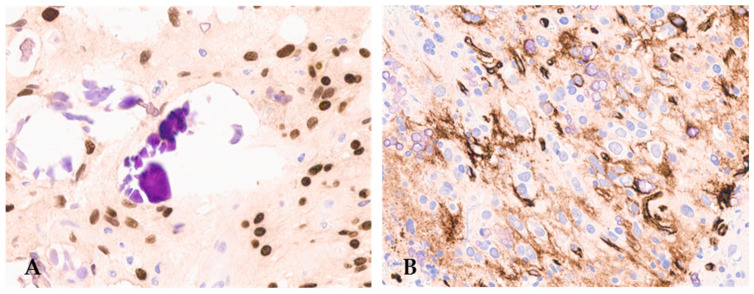
F3T3 diffuse glioma is positive for OLIG2 (**A**) (original magnification, ×200) and displays extra-vascular immuno-reactivity for CD34 (**B**) (original magnification, ×200).

F3T3 gliomas also have peculiar immunohistochemical characteristics. In addition to GFAP and OLIG2 positivity in the tumor cells (Figure 3), as expected for a diffuse glioma, more than 50% of cases exhibit CD34 immunostaining in ramified cells or along the cell membrane (Figure 3) [12,20,27].

ATRX expression is typically retained, and P53 is positive in less than 10% of tumor cells [20], in accordance with the absence of mutations in *ATRX* or *TP53* in these tumors. Immunostaining for IDH1 p. R132H is consistently negative [20]. As mentioned above, strong immunoexpression of FGFR3 was reported in the majority of F3T3 gliomas and mainly in areas showing the typical recurrent morphological features, suggesting that neoplastic cells in these zones could have the highest activation of FGFR3 downstream signaling [20].

## 4. Molecular Features of Diffuse Gliomas with High-Grade Histology and *FGFR3*::*TACC3* Fusion

F3T3 gliomas typically lack *IDH1/2* mutations and 1p/19q codeletion [11,16,20,27], which is important for their differential diagnosis towards oligodendrogliomas. These tumors are also persistently *H3*-wildtype and lack *BRAF* p. V600E mutation [11,16,20]. 

Similar to GBM *IDH*-wildtype, over 80% of histologically high-grade F3T3 gliomas harbor concurrent gains of chromosome 7 and loss of chromosome 10 (+7/−10) and/or *pTERT* mutation [28]. Moreover, more than 50% of these tumors feature *CDKN2A/B* homozygous deletion [11]. Nonetheless, in contrast to GBM *IDH*-wildtype, *EGFR* amplification is rare in F3T3 gliomas. Among 197 cases across four different cohorts, only 11 (5.6%) had *EGFR* amplification [11,16,20,28], whereas this genetic alteration was detected in approximately 43% of GBMs of *IDH*-wildtype [11,16]. Additional molecular distinguishing features of GBM *IDH*-wildtype are significantly higher frequencies of *CDK4* and *MDM2* amplification in high-grade F3T3 gliomas (22% vs. 7% for *CDK4* amplification; 20% vs. 4% for *MDM2* amplification) [16], whereas *MGMT* promoter methylation is similarly frequent in the two tumors [16,28].

*PDGFRA*, *MET*, and *KIT* amplification do not appear to be part of the molecular portrait of high-grade F3T3 gliomas, as they were absent in a cohort of 36 cases [11].

Over the last ten years, there has been accumulating evidence that different tumors exhibit distinct DNA methylation profiles, depending on their cell of origin and the genetic alterations that they acquired during initiation and progression [29]. Therefore, DNA methylation profiling is currently used for CNS tumor classification and for identification of novel tumor types [29]. In practice, the DNA methylation profile of a neoplastic sample is compared with data from over 2800 tumors collected in the Brain Tumor Classifier of Heidelberg University (website: www.molecularneuropathology.org). The classifier algorithm is based on the random forest algorithm, and the main output is a classification score that indicates similarity to one of the included CNS tumor methylation classes [29]. A score from 0 to 1 is generated for every class, and a score above the cut-off of 0.90 indicates a match with the methylation class. Over the years, the Heidelberg classifier has been periodically updated, giving rise to different versions, with the most recent being version 12.8.

Of 66 F3T3 gliomas with high-grade histology, which were profiled for DNA methylation in three different studies, 54 matched the methylation class glioblastoma “*IDH*-wildtype” and 12 did not match any classes, using version 11b4 of the Heidelberg classifier [11,12,28]. A comparison between 34 high-grade F3T3 gliomas and 100 GBM *IDH*-wildtype cases lacking *FGF3*::*TACC3* fusion revealed that the former were more likely to be assigned the mesenchymal or RTK II subclass than the latter [11].

Notably, two different studies have highlighted the inconsistency of the methylation class for some F3T3 gliomas using different versions of the Heidelberg Classifier [12,28]. In the cohort studied by Wu et al. [28], six F3T3 gliomas matched the methylation class GBM *IDH*-wildtype in version 11b4 of the classifier. However, only one matched the same methylation class in version 12.5 of the classifier, whereas the other five did not match any methylation classes but achieved the highest score for GBM mesenchymal subclass in four cases and ganglioglioma in one [28]. In a separate cohort reported by Metais et al., four histologically high-grade F3T3 gliomas were classified as GBM *IDH*-wildtype in version 11b4 of the classifier. Two of these cases did not match any classes in version 12.5, and one of these had the highest score for ganglioglioma [12]. Although most histologically high-grade F3T3 gliomas do not match any methylation class in version 12.5 of the classifier, it should be noted that on t-distributed stochastic neighbor embedding (t-SNE), they tend to form a homogeneous cluster near the GBM mesenchymal subtype [12], which underlines the genetic and epigenetic similarity with GBM.

## 5. Molecular Features of Diffuse Gliomas with Low-Grade Histology and *FGFR3*::*TACC3* Fusion

About 3.5% of histologically low-grade diffuse gliomas harbor *FGFR3*::*TACC3* fusion [24], with approximately forty-nine cases reported to date across various studies [12,20,23,27,28,30,31,32,33,34,35,36,37] (Table 1).

We observed an additional case in a 68-year old woman (case 30 in Table 1). Considering 43 cases with available information on patient age, 11 were diagnosed in children or adolescents (<18 years) (Table 1).

Histologically low-grade F3T3 gliomas display genetic and epigenetic heterogeneity.

Molecular stigmata of GBM *IDH*-wildtype were observed in 69% of cases (28/41); 51% (21/41) harbored +7/−10, and 62.5% (25/40) showed *pTERT* mutations. *EGFR* amplification was present in only two of 41 cases (4.8%) (cases 37 and 45 in Table 1), both of which had *pTERT* mutation and +7/−10. Notably, the molecular features of GBM *IDH*-wildtype were found only in tumors from adult patients, whereas no tumors from patients under the age of 18 harbored these genetic alterations (*p* < 0.0001).

DNA methylation profiling was available in 24 of 28 cases with molecular alterations typical of GBM (Table 1). Using version 11b4 of the classifier, fourteen cases matched the methylation class GBM *IDH*-wildtype, whereas ten did not match any methylation class. Notably, when using version 12.5 of the classifier, no cases had a match with any classes, with the exception of one case that had no match in 11b4 and matched ganglioglioma in version 12.5 (case 23 in Table 1). However, this latter case had an adverse clinical course, and the patient had disease progression at 28 months and died after 66 months [12].

DNA methylation profiling was available for seven histologically low-grade F3T3 gliomas that lacked molecular stigmata of GBM (cases 21, 22, 24, 26, 27, 28, and 29 in Table 1). All these cases were from patients below 18 years of age, with the exception of one case from a 29-year-old man (case 27 in Table 1). Two cases, in a 12-year-old and a 6-year-old patient, matched ganglioglioma or disembryoplastic neuroepithelial tumor (DNET), using either version 1b4 or 12.5 of the classifier (cases 22 and 28 in Table 1) [12]. Four cases did not match any methylation classes in both versions of the classifier (cases 21, 24, 26, 29); however, they reached the highest score for a glioneuronal tumor methylation class [12]. The remaining case (case 25 in Table 1) did not match any classes in version 11b4 of the classifier but matched ganglioglioma in version 12.5 [12]. 

Although most of the 15 histologically low-grade F3T3 gliomas analyzed by Metais et al. [12] did not match any methylation class in version 12.5 of the classifier, t-SNE DNA methylation profiling data analysis showed that they were distributed in three clusters [12]. One was formed of tumors, exclusive to adults, that displayed molecular features of GBM *IDH*-wildtype and were near the GBM mesenchymal subtype methylation class in the t-SNE plot (cases 16–20 in Table 1) [12]. These tumors may be precursors of histologically high-grade F3T3 gliomas. The second cluster (cases 21–25) comprised tumors, either in adults or in children, that either had or lacked molecular alterations of GBM *IDH*-wildtype and were near ganglioglioma in the t-SNE plot [12]. Finally, the third cluster included four tumors that, expect for one case, were diagnosed in children, lacked molecular stigmata of GBM and were near DNET in the t-SNE plot [12].

It should be noted that F3T3 gliomas lacking *pTERT* mutations, those having other *FGFR3*::*TACC3* fusions besides *FGFR3 (ex17)*::*TACC3 (ex11)*, or those resected from patients younger than 40 years exhibit a significantly better prognosis [12].

## 6. Histological Differential Diagnosis of F3T3 Gliomas

Due to their morphological characteristics, F3T3 gliomas may be histologically mistaken for other tumors. Oligodendroglioma is one of the primary differential diagnoses, as it also displays a diffuse growth pattern, clear cells, a thin capillary network, and microcalcifications [25]. However, oligodendroglioma is defined by the co-occurrence of mutations in the *IDH1* or *IDH2* genes and 1p/19q codeletion, which are absent in F3T3 gliomas. Therefore, in diffuse gliomas displaying tumor cells with roundish/ovoid nuclei and clear cytoplasm, examining *IDH* mutations is essential to eliminate oligodendroglioma as a potential diagnosis. Notably, the *FGFG3*::*TACC3* fusion was not found in any oligodendrogliomas *IDH*-mutant and 1p/19q codeleted, suggesting that this genetic alteration is exclusive to gliomas of astrocytic lineage [39]. 

Ganglioglioma may also be included in the differential diagnosis of F3T3 gliomas, as it shares characteristics such as desmoplasia, CD34 extra-vascular immunostaining, and microcalcifications [40]. This grade 1 glioneuronal tumor primarily occurs in the temporal lobe and is molecularly characterized by alterations in the MAPK pathway [40]. A crucial diagnostic feature of ganglioglioma is the presence of neoplastic ganglionic cells that lack NeuN immunoreactivity, distinguishing it from F3T3 diffuse gliomas. As previously mentioned, some histologically low-grade F3T3 gliomas display a DNA methylation profile matching the methylation class of ganglioglioma, as determined using version 12.5 of the Heidelberg classifier [12]. Furthermore, t-distributed stochastic neighbor embedding DNA-methylation profiling data analysis revealed a close proximity between at least some F3T3 gliomas and the ganglioglioma methylation class [12], suggesting epigenetic similarity between the two tumors. Although ganglioglioma may harbor mutations or fusions of *FGFR* genes, *FGFR3*::*TACC3* fusion has not been observed in any tumor with histological features of ganglioglioma [12].

The most challenging differential diagnosis for histologically low-grade F3T3 glioma involves PLNTY. This is a grade 1, *IDH*-wildtype, diffuse glioma arising in young individuals with seizures. It displays frequent oligodendroglioma-like components, calcifications, CD34 immunoreactivity, and a unique DNA methylation profile [41]. The WHO 2021 classification specifies the essential diagnostic criteria for PLNTY, which include the presence of the *BRAF* p. V600E mutation, *FGFR2* or *FGFR3* fusions, or other MAPK abnormalities [41]. The similarity in morphology and the inclusion of *FGFR3* fusions as a diagnostic criterion have likely led to confusion and overlap between PLNTY and F3T3 gliomas. However, to date, no histologically low-grade F3T3 gliomas profiled for DNA methylation have matched the methylation class of PLNTY [12,28]. In the original series of PLNTYs described by Huse et al., the only case harboring *FGF3*::*TACC3* fusion was not subjected to DNA methylation profiling [33]. These findings suggest that *FGFR3*::*TACC3* fusion is not a part of the molecular portrait of PLNTY and that PLNTYs reported to have *FGFR3*::*TACC3* fusion were likely misdiagnosed, because none of these cases was analyzed for DNA methylation profile to confirm a match to this tumor methylation class [30,31,37,42]. Furthermore, one case reported as PLNTY and harboring *FGFR3*::*TACC3* fusion displayed a clinical course atypical for PLNTY and underwent malignant transformation [30], reinforcing the notion that PLNTY and histologically low-grade F3T3 gliomas are distinct tumor types.

## 7. Conclusions

F3T3 gliomas do not constitute a distinct nosological tumor type according to WHO 2021 classification, due to their genetic and epigenetic heterogeneity.

Some of these tumors possess high-grade histopathological features similar to those of GBM and exhibit a DNA methylation profile consistent with GBM. However, they are characterized by a significantly better prognosis, suggesting that they may represent a distinct subtype of GBM.

The classification of histologically low-grade F3T3 gliomas is more complex, due to their higher genetic and epigenetic heterogeneity, which presents significant challenges in determining whether and in what circumstances they should be classified as low grade or high grade. A subset of histologically low-grade F3T3 gliomas, exclusive to adults, exhibits molecular features of GBM, including +7/−10 and/or *pTERT* mutation, and may represent precursors of high-grade F3T3 gliomas. DNA methylation profiling did not prove useful in classifying these tumors, as the majority failed to match any methylation classes, and one case matching ganglioglioma methylation class displayed an unfavorable clinical course. Furthermore, follow-up data for these cases are limited, which makes it difficult to establish whether these tumors should be classified as GBMs based on their molecular profile. However, t-SNE DNA methylation analysis showed that most of these tumors form a cluster near the GBM mesenchymal methylation class, suggesting genetic and epigenetic similarities between the two.

Another group of histologically low-grade F3T3 gliomas, almost exclusive to pediatric patients, lacks molecular alterations of GBM. Some of these cases match methylation classes of glioneuronal tumors, suggesting a similarity. It is currently unclear what the most appropriate diagnosis for these neoplasias is, but diffuse glioma with MAPK pathway alterations may be a possible option.

In summary, the majority of the existing literature pertains to high-grade F3T3 gliomas, with limited information available for low-grade cases. Further research is required to better understand the correlation between the molecular features, or age of onset, and the clinical aggressiveness of these tumors, with the aim of refining the grading system and determining the most appropriate treatment approach.

## Figures and Tables

**Table 1:** Molecular features of diffuse glioma cases with *FGFR3*::*TACC3* fusion reported to date.

Case [Refs.]	Age	Sex	+7/−10	*pTERT*	*EGFR* Ampl	MC	t-SNE Cluster	Resection	Adjuvant Therapy	FU	Length (Mo)
1 [30]	15	F	no	NA	no	no match	NA	gross total	no	alive	34
2 [20]	74	F	yes	mut	no	NA	NA	biopsy	NA	NA	NA
3 [20]	59	F	yes	mut	no	NA	NA	gross total	NA	NA	NA
4 [20]	72	F	no	wt	no	NA	NA	gross total	NA	NA	NA
5 [31]	14	F	NA	NA	NA	NA	NA	NA	NA	NA	NA
6 [32]	NA	M	no	wt	no	NA	NA	NA	NA	NA	NA
7 [32]	NA	F	no	wt	no	NA	NA	NA	NA	NA	NA
8 [33]	17	F	no	wt	no	NA	NA	gross total	NA	alive	89
9 [34]	NA	NA	NA	NA	NA	NA	NA	NA	NA	NA	NA
10 [34]	NA	NA	NA	NA	NA	NA	NA	NA	NA	NA	NA
11 [34]	NA	NA	NA	NA	NA	NA	NA	NA	NA	NA	NA
12 [35]	NA	NA	NA	NA	NA	NA	NA	NA	NA	NA	NA
13 [27]	53	F	yes	mut	no	NA	NA	partial resection	yes	alive	25
14 [38]	66	F	no	mut	no	NA	NA	biopsy	yes	dead	12
15 [38]	28	F	no	wt	no	NA	NA	gross total	no	alive	14
16 [12]	65	NA	yes	mut	no	GBM IDH-wt (v 11b4);	GB	NA	NA	NA	NA
						no match (v 12.5)					
17 [12]	56	NA	no	mut	no	no match	GB	NA	NA	NA	NA
						(v 11b4; v 12.5)					
18 [12]	77	NA	yes	mut	no	no match	GB	NA	NA	NA	NA
						(v 11b4; v 12.5)					
19 [12]	45	NA	no	mut	no	no match	GB	NA	yes	dead	11
						(v 11b4; v 12.5)					
20 [12]	24	NA	yes	mut	no	no match	GB	NA	NA	NA	NA
						(v11b4; v 12.5)					
21 [12]	4	NA	no	wt	no	no match	GG	NA	NA	NA	NA
						(v 11b4; v 12.5)					
22 [12]	12	NA	no	wt	no	GG (v 11b4);	GG	NA	NA	NA	NA
						GG (v 12.5)					
23 [12]	72	NA	no	mut	no	no match (v 11b4);	GG	NA	yes	dead	66
						GG (v 12.5)					
24 [12]	10	NA	no	wt	no	no match (v 11b4);	GG	NA	NA	NA	NA
						GG (v 12.5)					
25 [12]	38	NA	yes	mut	no	no match (v 11b4);	GG	NA	NA	NA	NA
						GG (v 12.5)					
26 [12]	13	NA	no	wt	no	no match	DNET	NA	NA	NA	NA
						(v11b4; v 12.5)					
27 [12]	29	NA	no	wt	no	no match	DNET	NA	yes	alive	37
						(v 11b4; v 12.5)					
28 [12]	6	NA	no	wt	no	LGG, DNET (v 11b4) DNET (v 12.5)	DNET	NA	NA	NA	NA
29 [12]	1	NA	no	wt	no	no match	DNET	NA	NA	NA	NA
30	68	F	yes	mut	no	GBM (v 11b4)	NA	gross total	yes	alive	14
						no match (v 12.5)					
31 [36]	6	M	NA	NA	NA	NA	NA	NA	NA	NA	NA
32 [37]	57	M	NA	NA	NA	NA	NA	NA	NA	alive	12
33 [23]	16	F	NA	NA	no	NA	NA	NA	NA	NA	NA
34 [23]	54	M	NA	NA	no	NA	NA	NA	NA	NA	NA
35 [28]	71	F	yes	mut	no	no match (v 11b4)	NA	NA	yes	alive	69
36 [28]	74	M	yes	wt	no	GBM (v 11b4)	NA	NA	yes	dead	20
						no match (v 12.5)					
37 [28]	68	F	yes	mut	yes	no match (v 11b4)	NA	NA	yes	dead	23
38 [28]	58	F	yes	mut	no	no match (v 11b4)	NA	NA	yes	dead	39
39 [28]	70	M	yes	mut	no	no match (v 11b4)	NA	NA	yes	dead	10
40 [28]	63	M	yes	wt	no	no match (v 11b4)	NA	NA	yes	alive	70
41 [28]	59	M	no	mut	no	GBM (v 11b4)	NA	NA	yes	alive	34
42 [28]	72	M	yes	mut	no	GBM (v 11b4)	NA	NA	yes	alive	65
43 [28]	64	M	yes	mut	no	GBM (v 11b4)	NA	NA	NA	NA	NA
44 [28]	70	F	no	mut	no	GBM (v 11b4)	NA	NA	NA	dead	1
45 [28]	47	F	yes	mut	yes	GBM (v 11b4)	NA	NA	NA	dead	1
46 [28]	73	M	yes	wt	no	GBM (v 11b4)	NA	NA	NA	dead	14
47 [28]	NA	F	no	mut	no	no match (v 11b4)	NA	NA	NA	dead	NA
48 [28]	60	F	yes	mut	no	no match (v 11b4)	NA	NA	NA	NA	NA
49 [28]	62	F	yes	mut	no	GBM (v 11b4)	NA	NA	NA	dead	37
50 [28]	58	M	yes	mut	no	no match (v 11b4)	NA	NA	NA	dead	14

Ampl: amplification. MC: methylation class. Mo: months. F: female. M: male. NA: not available. GBM: glioblastoma. GG: ganglioglioma. LGG: low-grade glioma. DNET: disembryoplastic neuroepithelial tumor.
